# Metastatic Clear Cell Renal Cell Carcinoma Identified in a Gastric Biopsy: Diagnostic Recognition in an Unusual Site

**DOI:** 10.7759/cureus.102416

**Published:** 2026-01-27

**Authors:** Yanina Nikolaus, Saroj Sigdel

**Affiliations:** 1 Pathology and Laboratory Medicine, Marshall University Joan C. Edwards School of Medicine, Huntington, USA

**Keywords:** clear cell renal carcinoma, gastric metastasis, immunohistochemical panel, metastatic rcc, pax8, renal cell carcinoma (rcc)

## Abstract

Metastatic clear cell renal cell carcinoma (ccRCC) to the stomach is rare and may mimic other clear cell neoplasms when encountered in limited biopsy samples. We report a case in which a gastric biopsy provided the first histopathologic confirmation of metastatic ccRCC in a 65-year-old man who had not yet undergone tissue sampling of a suspected renal lesion. The biopsy showed nests of epithelioid cells with clear cytoplasm and delicate vasculature, and immunohistochemical (IHC) staining demonstrated strong positivity for paired box gene 8 (PAX8), renal cell carcinoma (RCC) marker, and CD10, supporting renal origin. Negative staining for cytokeratin 7 (CK7), cytokeratin 20 (CK20), caudal-type homeobox transcription factor 2 (CDX2), and thyroid transcription factor 1 (TTF-1) helped exclude primary gastric, gastrointestinal, and pulmonary neoplasms. Subsequent imaging confirmed a renal mass concordant with the IHC findings. This case underscores the importance of recognizing morphologic clues and using a targeted IHC panel when evaluating clear cell neoplasms in the gastrointestinal tract.

## Introduction

Clear cell renal cell carcinoma (ccRCC) commonly metastasizes to the lungs, bone, and liver; however, metastasis to the stomach is exceedingly uncommon. When encountered in a limited biopsy sample, metastatic ccRCC may resemble other clear cell neoplasms, presenting a diagnostic challenge [1-3]. Accurate identification is crucial, particularly when metastatic involvement is detected before tissue sampling of a suspected renal mass.

This report describes a rare instance in which a gastric biopsy served as the initial diagnostic confirmation of metastatic ccRCC and highlights the histomorphologic and immunohistochemical (IHC) features that facilitate distinction from primary gastric tumors and other metastatic clear cell malignancies.

## Case presentation

A 65-year-old man with a past medical history significant for gastroesophageal reflux disease presented to the emergency department with several days of progressive left flank and left lower quadrant abdominal pain, initially worsened with respiration and later constant in nature. His symptoms were accompanied by abdominal distension, nausea, vomiting, frequent belching, and constipation.

On presentation, he was tachycardic and found to have new-onset atrial fibrillation with rapid ventricular response. Laboratory evaluation demonstrated leukocytosis, hyponatremia, and hematuria (Table 1).

**Table 1:** Pertinent laboratory results at presentation

Laboratory Test	Patient Value	Reference Range
White blood cell count (WBC)	19.7 ×10³/µL	4.0–11.0 ×10³/µL
Serum sodium	128 mmol/L	135–145 mmol/L
Urinalysis – red blood cells (microscopic)	21–50 /HPF	0–2 /HPF

Computed tomography (CT) of the abdomen and pelvis revealed a large enhancing mass involving the inferior pole of the left kidney, radiographically consistent with renal cell carcinoma, with additional findings concerning for metastatic disease, including retroperitoneal lymphadenopathy, pulmonary nodules, hepatic lesions, and a luminal mass within the gastric body. Given the gastric lesion identified on imaging, gastroenterology was consulted for further evaluation.

**Figure 1:**
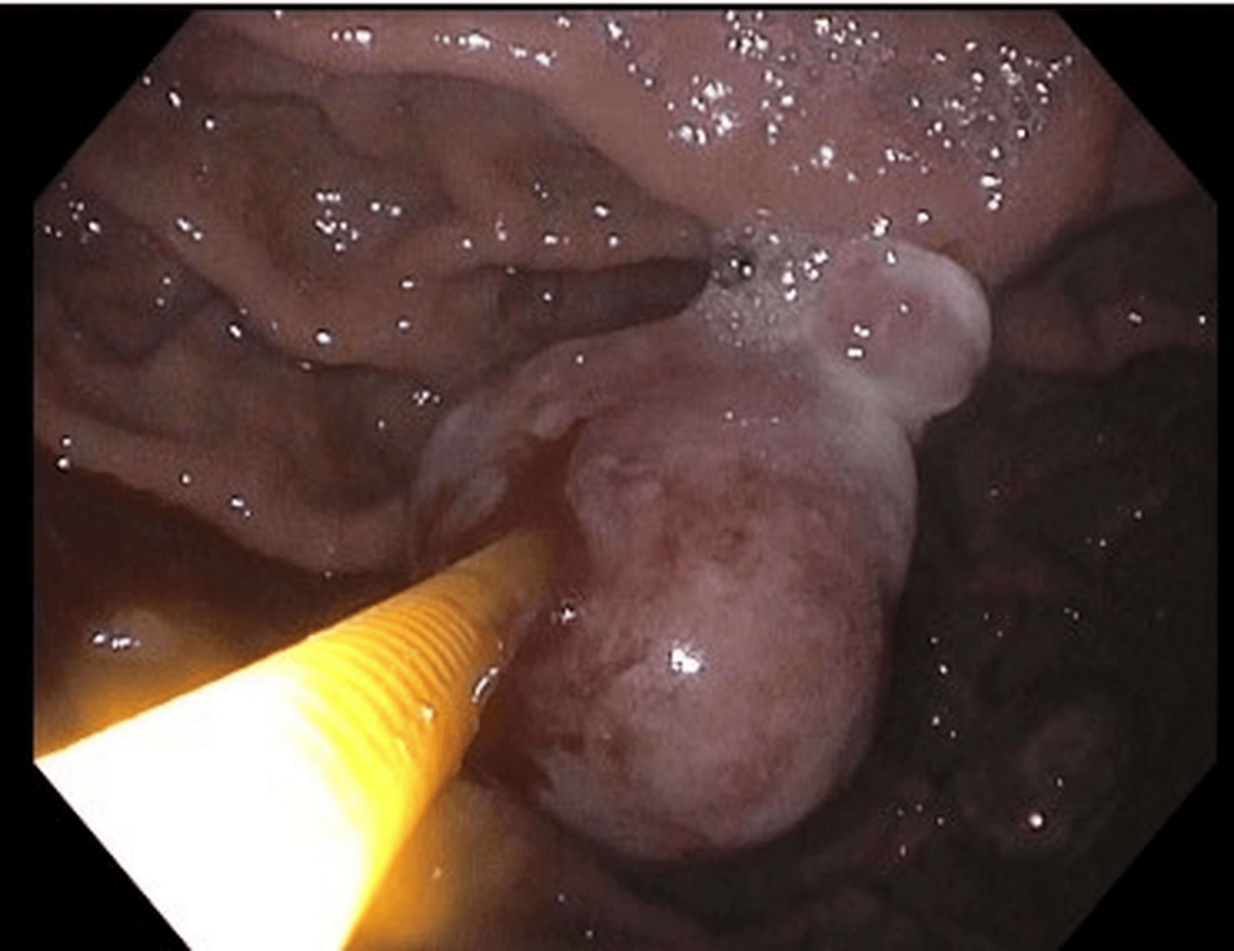
Endoscopic evaluation demonstrating an ulcerated, exophytic mass

**Figure 2:**
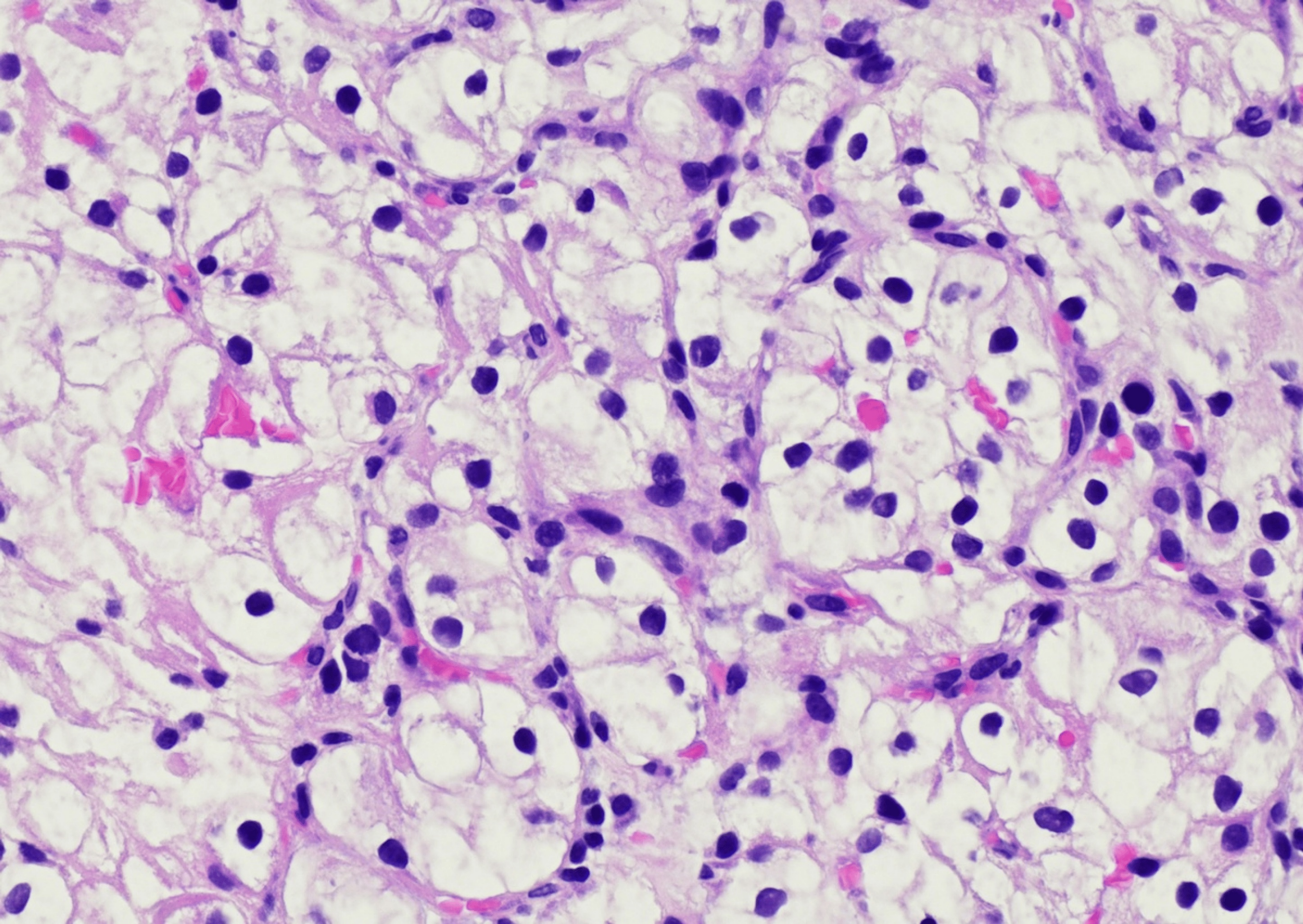
Histologic features of the gastric lesion (H&E, 40×) Clear cell tumor nests with distinct borders, prominent nucleoli, and delicate vasculature.

Endoscopic evaluation revealed an ulcerated, exophytic mass in the gastric body (Figure 1), and biopsies of the lesion were obtained. At the time of endoscopic evaluation, cross-sectional imaging of the abdomen was also available for clinical assessment. Histologic examination of the gastric biopsy demonstrated nests of clear to finely granular epithelioid cells with distinct cell borders, prominent nucleoli, and a delicate branching vasculature, features characteristic of metastatic ccRCC. No native gastric mucosa was identified (Figure 2).

**Figure 3:**
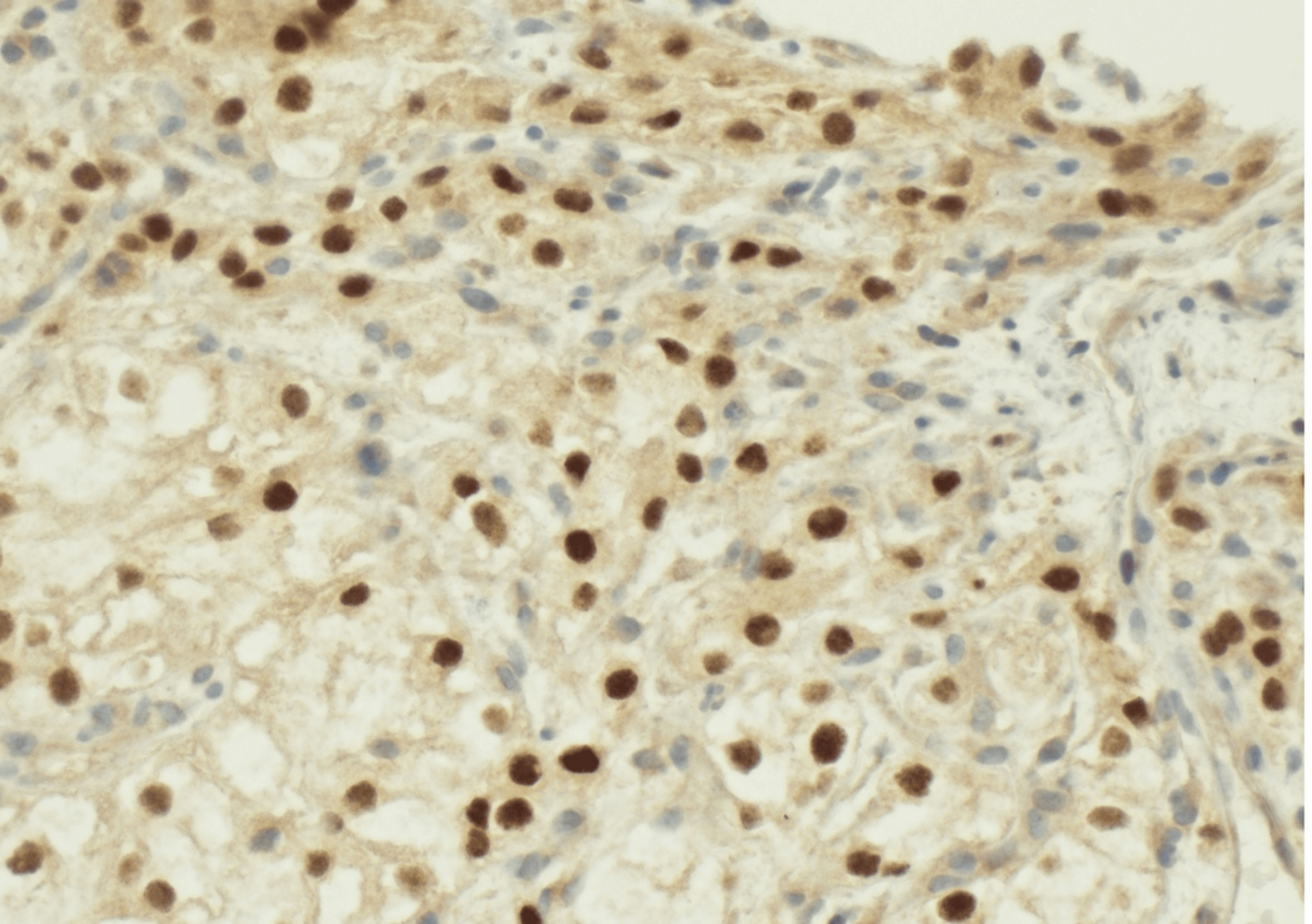
PAX8 immunohistochemistry confirming renal origin (40×) Diffuse nuclear PAX8 expression in tumor cells. PAX8: paired box gene 8

A focused immunohistochemical (IHC) panel was performed on the gastric biopsy specimen (Table 2). The tumor cells showed strong positivity for AE1/AE3, renal cell carcinoma (RCC) marker, CD10, and paired box gene 8 (PAX8), with negative staining for cytokeratin 7 (CK7), cytokeratin 20 (CK20), caudal-type homeobox transcription factor 2 (CDX2), and thyroid transcription factor 1 (TTF-1). Diffuse nuclear PAX8 expression supported renal origin (Figure 3).

**Table 2:** Immunohistochemical profile of the gastric biopsy Immunohistochemical staining results supporting metastatic clear cell renal cell carcinoma (ccRCC). Positivity for PAX8, RCC marker, CD10, and AE1/AE3 confirms renal epithelial origin, while negative staining for CK7, CK20, CDX2, and TTF-1 excludes primary gastric, intestinal, and pulmonary neoplasms. RCC: renal cell carcinoma; PAX8: paired box gene 8; CK7: cytokeratin 7; CK20: cytokeratin 20; CDX2: caudal-type homeobox transcription factor 2; TTF-1: thyroid transcription factor 1

Marker	Result
AE1/AE3	Positive
RCC marker	Positive
CD10	Positive
PAX8	Positive (nuclear)
CK7	Negative
CK20	Negative
CDX2	Negative
TTF-1	Negative

**Figure 4:**
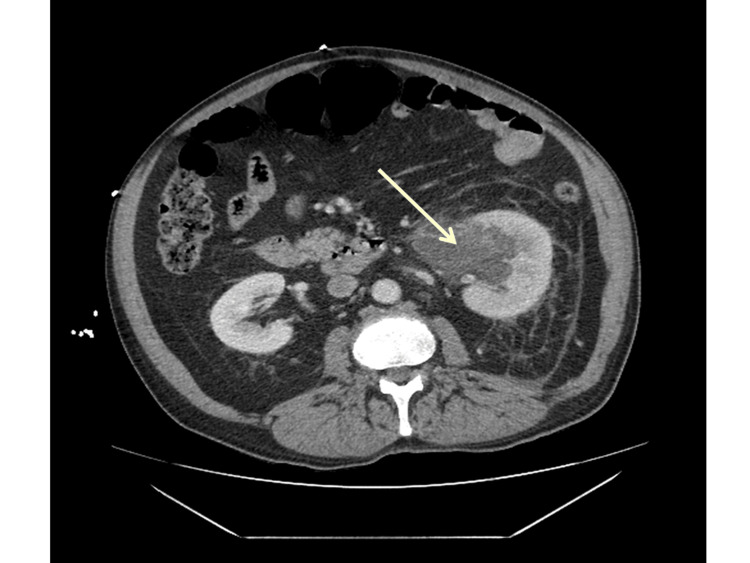
Contrast-enhanced CT (axial view) demonstrating a left renal mass (arrow), radiographically consistent with clear cell renal cell carcinoma.

Review of contrast-enhanced CT of the abdomen demonstrated a left renal mass radiographically consistent with ccRCC (Figure 4), supporting the interpretation of the gastric lesion as a metastatic focus. The pathologic findings were communicated to the treating clinical team. Subsequent staging confirmed widespread metastatic disease, including pulmonary, hepatic, and intracranial involvement. The patient was initiated on systemic therapy with pembrolizumab and lenvatinib and remains under active oncologic management.

## Discussion

ccRCC infrequently metastasizes to the gastrointestinal (GI) tract, and gastric involvement is particularly rare, with most reports limited to isolated case reports and small series [4-7]. Larger clinicopathologic series and reviews have further characterized the spectrum of GI metastases from ccRCC and confirmed the rarity of gastric involvement [8-11]. When present, gastric metastases may mimic primary gastric tumors both endoscopically and histologically, underscoring the importance of recognizing key morphologic clues. The combination of clear cytoplasm, distinct cell borders, and a delicate arborizing vasculature, features well documented in metastatic ccRCC [4,5,8,9], should prompt consideration of renal origin, particularly when native gastric mucosa is absent, as in this case.

IHC plays a critical role in distinguishing metastatic ccRCC from primary gastric adenocarcinoma and other metastatic clear cell neoplasms. The immunoprofile observed here, positivity for AE1/AE3, RCC marker, CD10, and PAX8, with negative staining for CK7, CK20, CDX2, and TTF-1, is characteristic of renal origin and has been emphasized in prior reports as a reliable diagnostic pattern [1-3,5,9,10]. PAX8 in particular is a highly sensitive nuclear marker for renal epithelial differentiation and is rarely expressed in primary gastric tumors, making it especially valuable in limited biopsy specimens [12,13].

Recognition of these morphologic and immunophenotypic features in small GI biopsies is essential, as metastatic lesions may precede the diagnosis of the primary renal mass, as demonstrated in several prior cases and series [5,7-10]. Early and accurate identification of metastatic ccRCC ensures appropriate clinical staging and management, particularly when the GI tract serves as the initial site of presentation [8-11].

## Conclusions

Metastatic ccRCC to the stomach is exceedingly rare and may present diagnostic challenges, particularly when encountered on limited biopsy material without prior knowledge of a renal primary. Recognition of the characteristic morphologic features, clear cytoplasm, distinct cell borders, and a delicate vascular network, combined with a focused IHC panel, is essential for accurate diagnosis. The immunoprofile of PAX8, RCC marker, and CD10 positivity, with the absence of GI and pulmonary markers, provides strong evidence of renal origin. Early identification of metastatic ccRCC in unusual sites such as the stomach facilitates appropriate staging and clinical management, underscoring the critical role of pathology in guiding patient care.
